# Pterostilbene mitigates the senescence of human dermal fibroblast cells by enhancing mitochondrial quality

**DOI:** 10.3389/fphar.2025.1732154

**Published:** 2025-12-08

**Authors:** Xinyu Zhou, Ning Wang, Jihua Wei, Guizhi Li, Zhengwei Lue, Chang Liu, Qi Bao, Zhe Feng, Minjie Zhang, Hu Huang, Yue Li, Jing Wang, Xiangnan Zhang

**Affiliations:** 1 State Key Laboratory of Advanced Drug Delivery and Release Systems, Zhejiang Key Laboratory of Neuropsychopharmacology, College of Pharmaceutical Sciences, Institute of Pharmacology and Toxicology, Zhejiang University, Hangzhou, China; 2 Jinhua Institute of Zhejiang University, Jinhua, China; 3 Proya Cosmetics CO., LTD., Hangzhou, China; 4 School of Pharmacy, Hangzhou Medical College, Hangzhou, China; 5 Department of Plastic and Reconstructive Surgery, The Second Affiliated Hospital, School of Medicine, Zhejiang University, Hangzhou, China

**Keywords:** human dermal fibroblasts cells (HDFs), skin aging, senescent, pterostilbene, mitochondria, mitophagy

## Abstract

**Introduction:**

Pterostilbene (PT), a natural polyphenol found in blueberries and several grape varieties, exhibits pleotropic pharmacological effects. PT reduced the makers of aging caused by either ultraviolet (UV) light exposure or chemical stress in keratinocytes, whereas its potential anti-aging effects and underlying mechanisms in the dermis have not been elucidated.

**Methods:**

The anti-senescence effects of PT were investigated in human dermal fibroblasts (HDFs) using models of UVB-induced acute oxidative stress and replicative senescence. Key assays included senescence-associated beta-galactosidase (SA-β-gal) activity, RT-PCR, western blotting, immunofluorescence, live-cell confocal imaging with fluorescent probes, flow cytometry and mitochondrial respiration analysis. A mouse model of UVB-induced skin damage was used to evaluate PT’s anti-aging effects in vivo through histopathological examination and western blot analysis.

**Results:**

PT treatment mitigated senescence in HDFs, as shown by reduced SA-β-gal activity, p16, and p21, along with increased collagen expression. It restored mitochondrial morphology, MMP, and reduced mitochondrial reactive oxygen species in both senescent models. Furthermore, PT improved mitochondrial basal respiration, ATP production, and maximal respiration. Mechanistically, PT promoted mitophagy, indicated by enhanced TOM20/LC3 colocalization. In vivo, topical PT restored collagen, dermal thickness, and LC3, while reducing p21 levels in UVB-exposed mice.

**Discussion:**

Our findings demonstrate that PT delays dermal senescence by enhancing mitochondrial quality via enhancing mitophagy. These results highlight PT as a promising anti-aging agent capable of countering both intrinsic and extrinsic aging in the dermis.

## 1 Introduction

Being the outermost protective layer of the body, the skin is a vital organ responsible for protecting against external stimuli. Both intrinsic and extrinsic factors contribute to skin aging, leading to epidermis and dermis thinning, collagen and elastin degradation, and compromised barrier function (Csekes and Rackova, 2021; Thau et al., 2025; Zhang et al., 2024). Aged skin typically appears wrinkling, sagging and aberrant pigmentation (Chin et al., 2023), and is considered a risky factor for skin cancer (Banach et al., 2021; D'Uva et al., 2018). Skin aging involves multilayer alterations in both the epidermis and dermis (Nan et al., 2025). While the epidermal layer serves primarily as a protective barrier against external damage, the structural and functional decline of the dermis plays a more critical role in overall skin aging (Jipu et al., 2025).

As the main cell type in the dermis, fibroblasts are mesenchymal cells essential for maintaining both structural and functional homeostasis of skin. Fibroblasts regulate the synthesis, degradation, and remodeling of the extracellular matrix (ECM), which provides mechanical strength, elasticity, and hydration to the skin (Plikus et al., 2021; Thulabandu et al., 2018). The functional decline of fibroblasts in aging process directly leads to ECM metabolic imbalance, manifested as loss of collagen and elastin. These changes lead to aging phenotypes including wrinkles and skin laxity (Dorf and Maciejczyk, 2024; Freitas-Rodriguez et al., 2017). Therefore, developing effective strategies to counter the negative effects of fibroblast senescence is crucial for understanding and intervening in skin aging.

Pterostilbene (trans-3,5-dimethoxy-4′-hydroxystilbene, PT) is a natural polyphenol derived from blueberries and grapes (Nagarajan et al., 2022). Previous studies have shown that PT protects against UV-induced photo-damage through Nrf2/ARE pathway in human keratinocytes (Li et al., 2017) and exhibits anti-aging effects in sebaceous gland cells (Zhong et al., 2025). However, the protective effect of PT on dermal fibroblast senescence, as well as its underlying mechanisms, remains largely unclear.

Mitochondrial are critical organelles in maintaining cellular homeostasis beyond its role as power plant. Mitochondrial dysfunction has been listed as one of the markers of cellular aging (Kroemer et al., 2025). Decreased oxidative phosphorylation and mitochondrial membrane potential as well as excessive accumulation of reactive oxygen species (ROS) are key features of mitochondrial dysfunction in senescent cells (Song et al., 2023; van der Rijt et al., 2020). Substantial evidence indicates that mitochondrial quality decline is a key driver of skin cell aging. As a highly proliferative tissue, the skin relies on continuous epidermal regeneration, a process that demands substantial Adenosine triphosphate (ATP) supply, with mitochondria serving as the primary source of cellular energy (Yokouchi and Kubo, 2018; Zhang et al., 2023). Furthermore, ultraviolet radiation (UVR) induces mtDNA loss in dermal fibroblasts, accelerating premature skin aging (Krutmann and Schroeder, 2009). Therefore, maintaining the mitochondrial function and integrity is essential for delaying skin aging.

PT enhances mitochondrial function across diverse cellular models. PT enhances mitochondrial biogenesis and respiration in intestinal porcine enterocytes and adipocytes (Chen et al., 2021; Koh et al., 2023). PT also attenuates microglial inflammation and brain injury in intracerebral hemorrhage mice by correcting mitochondrial dynamics (Wu et al., 2023). Furthermore, PT ameliorates cellular aging by activating sirtuins and mitochondrial unfolded protein response (UPR^mt^) in neurons (Suarez-Rivero et al., 2022). Despite the facts that PT improves mitochondrial quality in a variety of cells, it remains not fully explored whether the improved the mitochondrial quality underlies its anti-aging effects on skin.

## 2 Materials and methods

### 2.1 Chemical and reagents

Pterostilbene (PT) (purity≥99%) was provided by Proya Cosmetics Co., Ltd. (Hangzhou, China). The PT was dissolved in dimethyl sulfoxide (DMSO) to make a stock solution of 400 mM. A PT cream (0.01%) and its corresponding vehicle cream were formulated by the same provider. The composition of the vehicle cream was as follows: Water, Glycerin, Caprylic/Capric Triglyceride, Cetearyl Alcohol, Glyceryl Stearate, Sodium Acrylate/Sodium Acryloyldimethyl Taurate Copolymer, 1,2-Hexanediol, Hydroxyacetophenone, Polyethylene glycol-100 Stearate, Isohexadecane, Polysorbate 80, Acrylates/C10-30 Alkyl Acrylate Crosspolymer, Tromethamine, ethylenediaminetetraacetic acid disodium, and Sorbitan Oleate.

### 2.2 Cell culture

Primary cultured HDFs were purchased from Guangdong BioCell Biotech Co., Ltd. (cat. no. PC 2031; lot no. 230131), which were derived from the skin of a patient between 8 and 10 years old. The cells were cultured in Dulbecco’s Modified Eagle Medium complete medium (DMEM; Gibco, 31600034) containing with 10% fetal bovine serum (FBS; Excell Bio, FSD500) and 1% penicillin-streptomycin (YEASEN, 60162ES76) in a 37 °C incubator with 5% CO_2_.

### 2.3 Establishment and treatment of UVB-induced acute oxidative stress model in HDFs

HDFs were divided into the following groups: the Control group, the UVB model group, and the PT treatment groups. Cells in the PT treatment group were pretreated with PT for 24 h, whereas both the Control and UVB model groups received the DMSO at the same final concentration. For UVB irradiation, the 6-well plate with cells in phosphate-buffered saline (PBS) were placed in the ultraviolet illuminometer and the program is set (Power: 0.30 mW/cm^2^; Time: 10 min; Energy: 180 mJ/cm^2^). After the program finished, the cells were normally cultured for 24 h and then detected.

### 2.4 Establishment and treatment of HDFs replicative senescence model

Young HDFs controls were defined as passages 5–10 (P5-10) cells from the primary cultured HDFs with minimal aging characteristics. Replicative senescence was achieved by serial passaging. Passages 35–40 (P35-40) HDFs were used as the senescent cells. During serial passage, PT was added to the culture medium to continuously treat the cells. The senescent group cells were maintained with the same concentration of DMSO throughout the passaging process.

### 2.5 Cell viability assay

HDFs were seeded at 8,000 cells/well in 96-well plates, treated for 24 h, and incubated with the CCK-8 reagent (APExBIO, K1018). The absorbance was measured at 450 nm.

### 2.6 Senescence-associated β-galactosidase (SA-β-gal) staining

SA-β-Gal staining was performed using an SA-β-Gal Staining Kit (Beyotime Biotechnology, C0602) according to the manufacturer instructions. Cells were fixed and incubated with the staining solution overnight at 37 °C overnight in a dry incubator without CO_2_. The blue SA-β-Gal-positive cells were imaged and quantified using a bright field microscope.

### 2.7 Real-time PCR

Total RNA was extracted using FreeZol Reagent (Vazyme, R711) and reverse-transcribed into cDNA using HiScript Ⅳ All-in-One Ultra RT SuperMix for qPCR (Vazyme, R433) according to the manufacturer’s instructions. Real-time PCR was performed using Taq Pro Universal SYBR qPCR Master Mix (Vazyme, Q712) on an Applied Biosystems™ QuantStudio™ 3 System (Thermo Fisher, ISO13485). Gene expression was normalized to GAPDH and the results were calculated using the 2−^ΔΔCt^ method. The primer sequences used for target gene expression identification were as follows: p16, sense 5′-TCG​CGA​TGT​CGC​ACG​GTA-3′ and anti-sense, 5′-CAT​CTA​TGC​GGG​CAT​GGT​TAC​TG-3’; GAPDH, sense 5′- GAA​AGC​CTG​CCG​GTG​ACT​AA-3′ and anti-sense 5′-GCA​TCA​CCC​GGA​GGA​GAA​AT-3’. All primers used for RT-qPCR were obtained from Shanghai GENEray Biotechnology Co., Ltd.

### 2.8 Western blot

Total proteins were extracted from cells and tissues using RIPA lysis buffer (NCM, WB3100) supplemented with protease and phosphatase inhibitor cocktail (Beyotime, P1045). Protein samples normalized by BCA assay were separated by SDS-PAGE and transferred to PVDF membranes (Milipore, ISEQ00010-1EA). The membranes were blocked with 5% skim milk for 2 h at room temperature. After that, the membranes were incubated overnight with the primary antibodies at 4 °C. The primary antibodies used were Rabbit Anti-p21 (ABclonal, A19094; 1:1,000), Rabbit Anti-LC3 Ⅱ (Sigma-Aldrich, I7543; 1:1,000), Rabbit Anti-TOM20 (ABclonal, A19403; 1:1,000), Rabbit Anti-GAPDH (Proteintech, 10494-1-AP; 1:10,000). Next, the membranes were incubated with HRP-conjugated secondary antibodies and then visualized using an enhanced chemiluminescence (ECL) system. The intensity of the bands was quantified using ImageJ software.

### 2.9 Immunofluorescence

15,000 cells per well grown on 24-well coverslips were fixed with 4% paraformaldehyde fix solution for 15 min, permeabilized with 0.2% Triton X-100 for 10 min, and blocked with 10% goat serum containing glycine for 30 min. Next, the cells were incubated overnight at 4 °C with the following primary antibodies: Rabbit Anti-Collagen Ⅰ (Proteintech, 14695-1-AP; 1:200), Rabbit Anti-Collagen Ⅲ (Abcam, ab7778; 1:200), Rabbit Anti-LC3Ⅱ (Proteintech, 14600-1-AP; 1:200), Mouse Anti-TOM20 (ABclonal, A27799; 1:200). Then the cells were washed with PBST and incubated with Alexa Fluor 488-conjugated goat anti-rabbit (ABclonal, AS053; 1:250) and Alexa Fluor 647-conjugated goat anti-mouse secondary antibodies (ABclonal, AS059; 1:250) for 1 h at room temperature. After a final wash with PBST, slides were mounted using Antifade Mounting Medium containing DAPI (Beyotime, P0131) for nuclear counterstaining. Images were captured using a fluorescence microscope.

### 2.10 Mitochondrial morphology and functional assays

- Mitochondrial morphology: Cells seeded in confocal microdishes were staining coincubated with 200 nM MitoTracker (Thermo Fisher Scientific, M22426) and Hoechst (APExBIO, K2407) for 30 min at 37 °C. After staining is complete replace the staining solution with fresh prewarmed media. Fluorescent images were acquired using an Olympus FV3000 confocal microscope with a ×100 oil objective.
- Membrane Potential: Cell suspension were staining incubated with 100 nM TMRM (Thermo Fisher Scientific, I34361) for 30 min at 37 °C. The cells were washed and resuspend with PBS. Analyze the cells on a flow cytometer. Median fluorescence intensity was calculated using FlowJo software.
- Mitochondrial ROS: Mitochondrial ROS levels were assessed using MitoSOX Red Mitochondrial Superoxide Indicator (YEASEN, 40778ES50). Cells were incubated with 5 µM MitoSOX in HBSS with Calcium and Magnesium (Solarbio, H1025) for 30 min. After staining is complete replace the staining solution with HBSS and observe cells using an Olympus FV3000 confocal microscope.

### 2.11 Mitochondrial respiration analysis

Mitochondrial respiration was analyzed using a Seahorse XF96 Extracellular Flux Analyzer (Agilent Technologies, 103,793–100), according to the manufacturer’s instructions. 15,000 HDFs per well were plated into the wells of an XF96 cell culture microplate and incubated at 37 °C in a CO_2_ incubator overnight to ensure attachment. The Oxygen consumption rate (OCR) involved sequential injection of 1.5 μM oligomycin, 2 µM FCCP, 0.5 μM rotenone and 0.5 μM antimycin-A. Protein normalization was performed after the assay.

### 2.12 Experiment animals and treatment protocols

Six-week-old male C57BL/6 mice were maintained under a 12 h light/dark cycle with free access to water and food. As previous described (Budluang et al., 2025; Choi et al., 2020), mice received localized UVB irradiation on depilated dorsal skin three times weekly for 4 weeks. The exposure dose began at 1 minimal erythemal dose (MED) and was increased weekly by 1 MED, reaching a final dose of 4 MED. The cream containing 0.01% Pterostilbene was evenly applied daily to the exposed dorsal skin of the mice during the experiment. All experiments were approved by and conducted in accordance with the ethical guidelines of the Zhejiang University Animal Experimentation Committee and were completely compliant with the National Institutes of Health Guide for the Care and Use of Laboratory Animals.

### 2.13 Hematoxylin and Eosin (HE) and Masson’s trichrome staining

Mice dorsal skin samples were fixed in 4% paraformaldehyde in PBS, then embedded in paraffin. Subsequently, the samples were sectioned and stained using HE and Masson’s trichrome for histopathologic examination. Dermal thickness and collagen density were quantified using the ImageJ software.

### 2.14 Statistical analysis

All statistical analyses were conducted using GraphPad Prism version 9.0. Student’s t-test was used for comparisons between two groups, whereas one-way analysis of variance (ANOVA) or two-way ANOVA was applied for multiple-group comparisons. Data are represented as means ± SD, and statistical significance was set at *p* < 0.05.

## 3 Results

### 3.1 PT attenuated UVB-induced acute oxidative stress in HDFs

**FIGURE 1:**
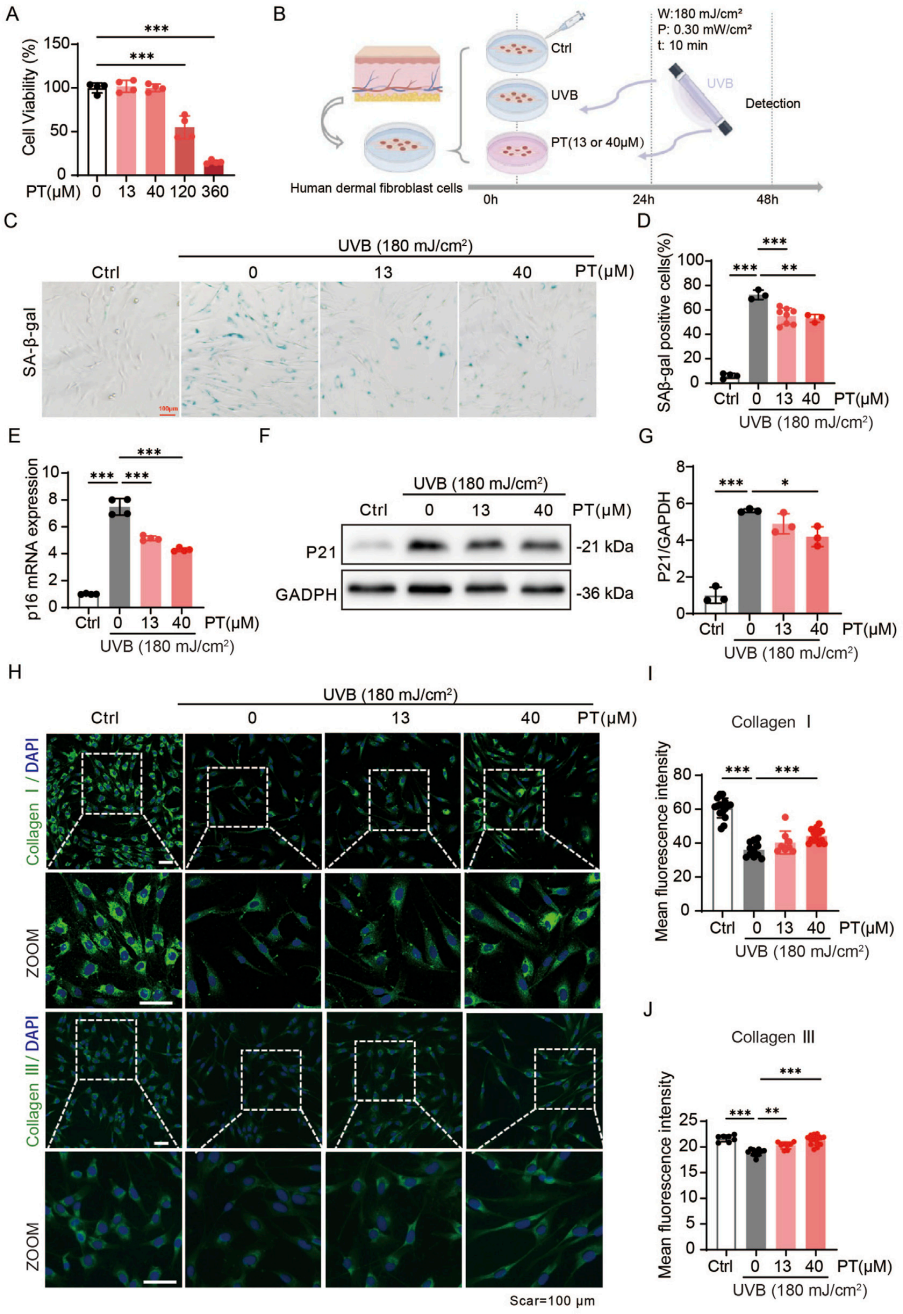
PT alleviated UVB-induced acute oxidative stress model phenotypes in HDFs. **(A)** Cell viability of HDFs pre-treated with different concentrations of PT for 24 h was measured by CCK8 assay. **(B)** Schematic diagram of the experimental procedure for establishing a UVB-induced acute oxidative stress model in HDFs. **(C,D)** Representative images of SA-β-gal staining showing the proportion of senescent cells (*n* = 3–8). Scale bar = 100 μm. **(E)** The mRNA expression of *p16* in UVB-induced HDFs (*n* = 4). **(F)** Western blot images and **(G)** quantification of p21 protein level in UVB-induced HDFs (*n* = 3). **(H)** Representative immunofluorescence images of Collagen Ⅰ and Ⅲ, respectively. **(I,J)** Quantification of the fluorescence intensity of Collagen Ⅰ and Ⅲ (*n* = 8–16). Data were shown as mean ± SD of three independent experiments. Statistical significance was determined by one-way ANOVA with Tukey’s multiple comparisons test. ***p < 0.001, **p < 0.01, *p < 0.05.

We initially verified the anti-aging effects of PT in HDFs. In a UVB-induced acute oxidative stress model established in HDFs, and following confirmation of non-cytotoxic PT concentrations (Figures 1A,B), PT treatment significantly attenuated UVB-induced cellular senescence. This was manifested as a concentration-dependent decrease in senescence-associated markers, including SA-β-gal, p16, and p21 levels (Figures 1C–G). Furthermore, PT restored the expression of collagen I and III (Figures 1H–J). These results indicated that PT attenuated UVB-induced acute oxidative stress in HDFs.

### 3.2 PT restored UVB-induced mitochondria dysfunction in HDFs

**FIGURE 2:**
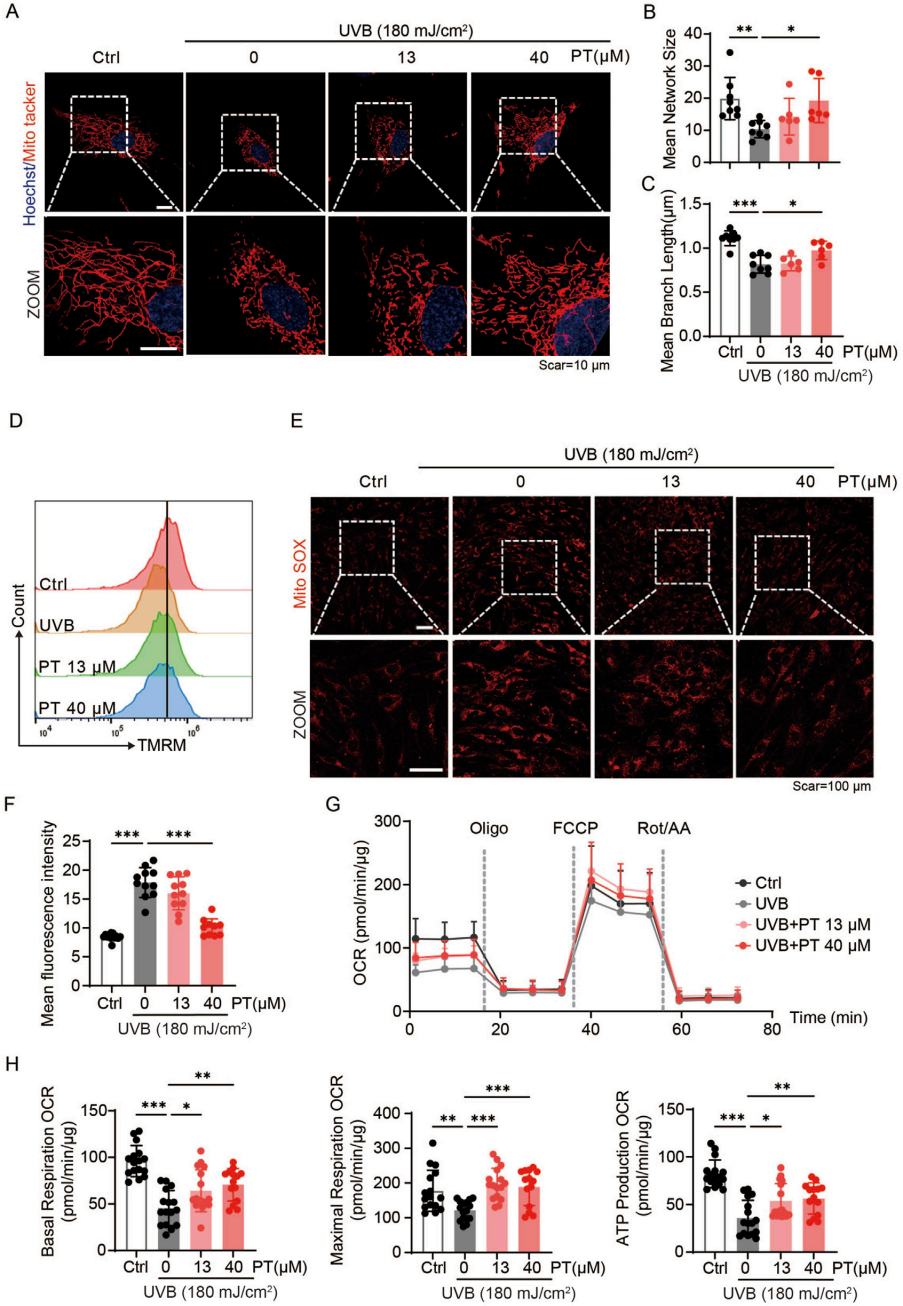
PT restored mitochondrial morphology and ameliorates mitochondrial dysfunction in UVB-induced HDFs. **(A)** Representative immunofluorescence images of mitochondrial labeled with MitoTracker Red in UVB-induced HDFs. Scale bar = 10 μm. **(B,C)** Quantification of mitochondrial morphology shown in **(A)** (*n* = 6–8). **(D)** Flow cytometry analysis of MMP measured by TMRM staining in UVB-induced HDFs. **(E)** Representative immunofluorescence images of mitochondrial ROS detected with MitoSOX in UVB-induced HDFs (*n* = 10–13). Scale bar = 100 μm. **(F)** Flow cytometry analysis of mitochondrial ROS. **(G)** Oxygen consumption rate (OCR) profiles representing mitochondrial respiration in HDFs (*n* = 14–16). **(H)** Quantificaiton of mitochondrial respiration parameters, including baseline respiration, ATP production, and maximal respiratory capacity of HDFs. Data were shown as mean ± SD of three independent experiments. Statistical significance was determined by one-way ANOVA with Tukey’s multiple comparisons test. ***p < 0.001, **p < 0.01, *p < 0.05.

To clarify whether PT protects against UVB-induced acute oxidative stress in HDFs by improving mitochondrial quality, both mitochondrial morphology and function were assessed. Mitochondria staining revealed that PT incubation restored the mitochondrial length and network size (Figures 2A–C), implying improved mitochondrial quality. Furthermore, flow cytometry analysis showed that PT prevented the loss of mitochondrial membrane potential by UVB exposure (Figure 2D). Consistently, MitoSOX fluorescence images revealed that PT suppressed UVB-induced mitochondrial ROS production, indicating improved respiratory efficiency (Figures 2E,F). To further quantify mitochondrial respiration, we assessed the oxygen consuming rate (OCR) in HFDs. The results demonstrated that 13 and 40 μM PT treatment significantly enhanced baseline respiration, ATP production, and maximal respiration compared to the UVB group (Figures 2G,H). Together, these results demonstrated that PT treatment ameliorated mitochondrial dysfunction in UVB-induced aged HDFs.

### 3.3 PT attenuated skin aging in replicative senescent HDFs

**FIGURE 3:**
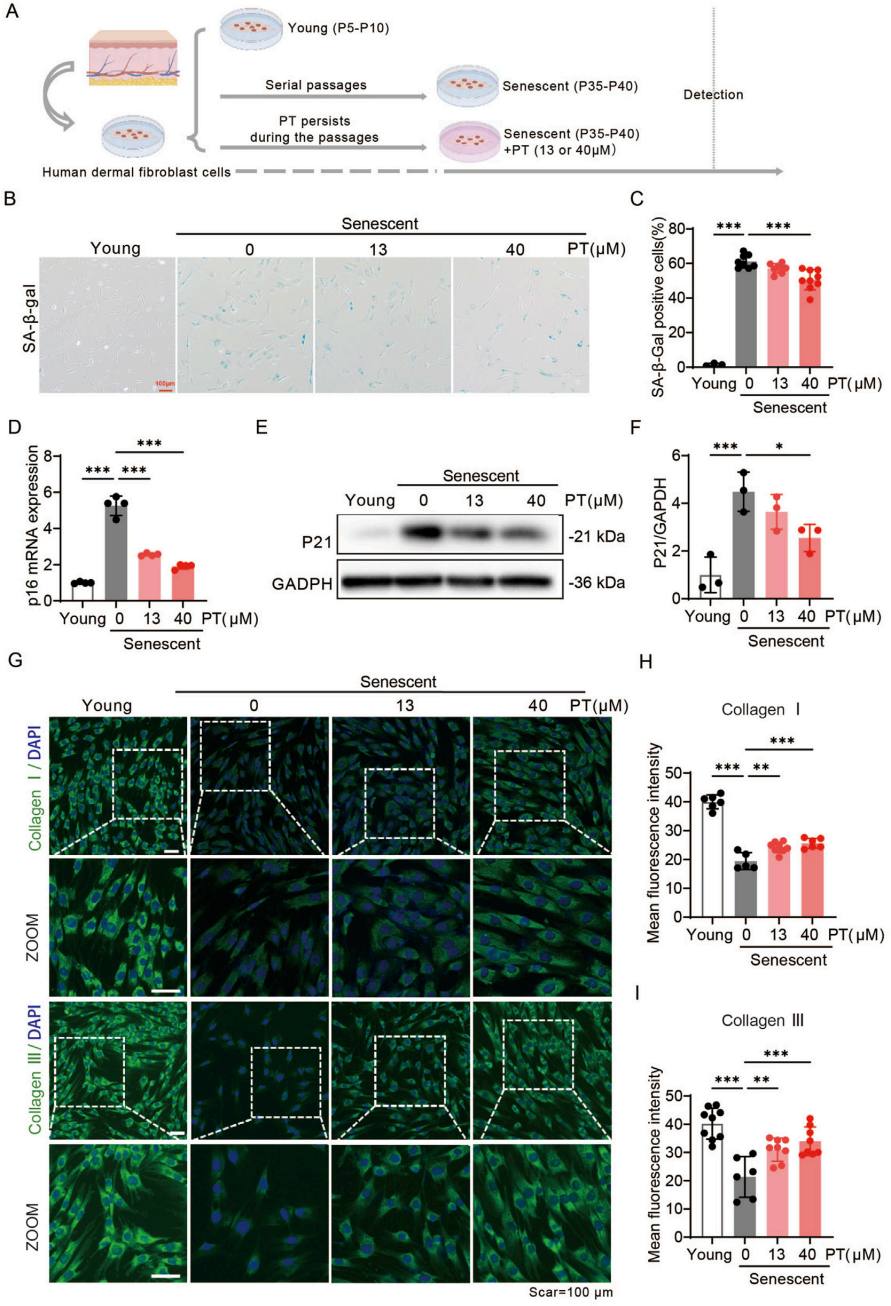
PT alleviated senescent phenotypes in replicative senescent HDFs. **(A)** Schematic diagram of the experimental procedure for establishing replicative senescent HDFs. **(B)** Representative images and **(C)** quantification of SA-β-gal staining showing the proportion of senescent cells (*n* = 3–9). Scale bar = 100 μm. **(D)** Relative mRNA levels of *p16* in replicative senescent HDFs (*n* = 4). **(E)** Western blot images and **(F)** quantification of p21 protein expression in UVB-induced HDFs (*n* = 3). **(G)** Representative immunofluorescence images of Collagen Ⅰ and Ⅲ, respectively. **(H,I)** Quantification of the fluorescence intensity of Collagen Ⅰ and Ⅲ (*n* = 6–9). Data were shown as mean ± SD of three independent experiments. Statistical significance was determined by one-way ANOVA with Tukey’s multiple comparisons test. ***p < 0.001, **p < 0.01, *p < 0.05.

To further verify the anti-aging effect of PT on the dermis, a replicative senescence model was employed by serially passaging the human foreskin-derived HDFs. PT was administrated continuously during the aging passage of HDFs (Figure 3A). HFDs within P4-P10 were defined as young HDFs, whilst those from passage P35-P40 were considered senescent HFDs. PT treatment significantly reduced cellular senescence in replicative HDFs, as evidenced by a concentration-dependent decrease in senescence-associated markers, including SA-β-gal, p16, and p21 levels (Figures 3B–F). As shown in Figures 3G–I, immunofluorescence images revealed that PT restored the expressions of Collagen Ⅰ and Collagen Ⅲ in a concentration-dependent manner. Collectively, these findings demonstrate that PT treatment attenuates replicative senescent HDFs, supporting its potential anti-aging effect on the dermis.

### 3.4 PT restored mitochondria dysfunction in replicative senescent HDFs

**FIGURE 4:**
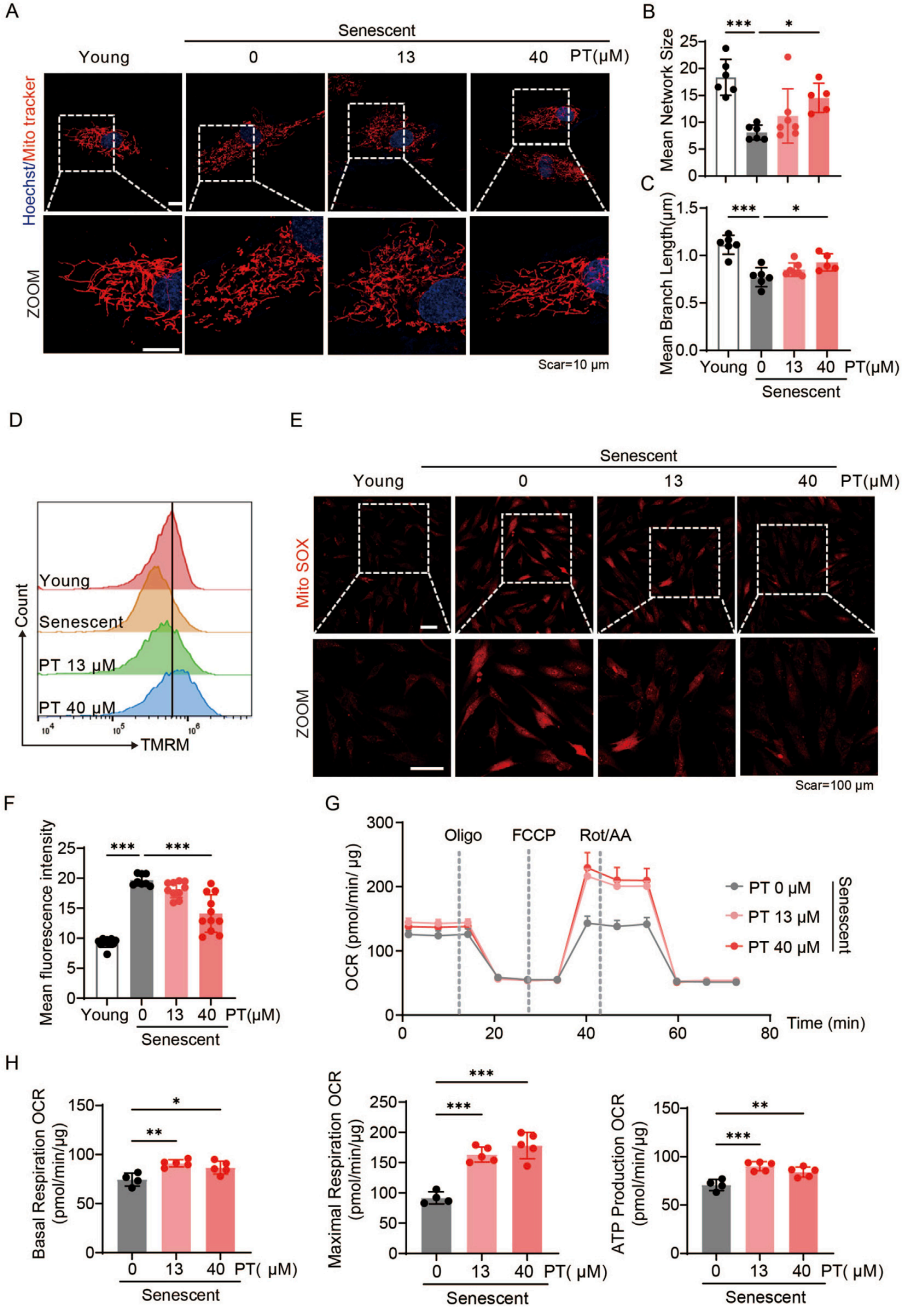
PT restored mitochondrial morphology and mitigated mitochondrial dysfunction in replicative senescent HDFs. **(A)** Representative immunofluorescence images of mitochondrial labeled with MitoTracker Red in replicative senescent HDFs. Scale bar = 10 μm. **(B,C)** Quantification of mitochondrial morphology shown in **(A)** (*n* = 5–7). **(D)** Flow cytometry analysis of MMP measured by TMRM staining in replicative senescent HDFs. **(E)** Representative immunofluorescence images of mitochondrial ROS detected with MitoSOX in replicative senescent HDFs. Scale bar = 100 μm. **(F)** Flow cytometry analysis of mitochondrial ROS (*n* = 8–11). **(G)** Oxygen consumption rate (OCR) profiles representing mitochondrial respiration in HDFs. **(H)** Quantificaiton of mitochondrial respiration parameters, including baseline respiration, ATP production, and maximal respiratory capacity of HDFs (*n* = 4–5). Data were shown as mean ± SD of three independent experiments. Statistical significance was determined by one-way ANOVA with Tukey’s multiple comparisons test. ***p < 0.001, **p < 0.01, *p < 0.05.

To investigate the effects of PT on mitochondrial quality in senescent HDFs, mitochondrial morphology, mitochondrial membrane potential, ROS levels and mitochondrial respiratory function were evaluated. As shown in Figures 4A–C, senescent HDFs exhibited reduced mitochondrial length and network size, along with decreased MMP, all of which were reversed by PT treatment (Figure 4D). Furthermore, MitoSOX immunofluorescence images revealed that senescent HFDs displayed significantly increased mitochondrial ROS compared to young group, senescent HDFs exhibited significantly increased mitochondrial ROS, an effect significantly attenuated by 40 μM PT treatment (Figures 4E,F). Mitochondrial respiration was assessed via oxygen consumption rate measurements. Specifically, PT treatment at 13 and 40 μM significantly enhanced baseline mitochondrial respiration, ATP production, and maximal respiration compared to the senescent group (Figures 4G,H). Our result demonstrated that PT treatment reversed mitochondrial dysfunction in replicative senescent HDFs.

### 3.5 PT promoted mitophagy in replicative senescent HDFs

**FIGURE 5:**
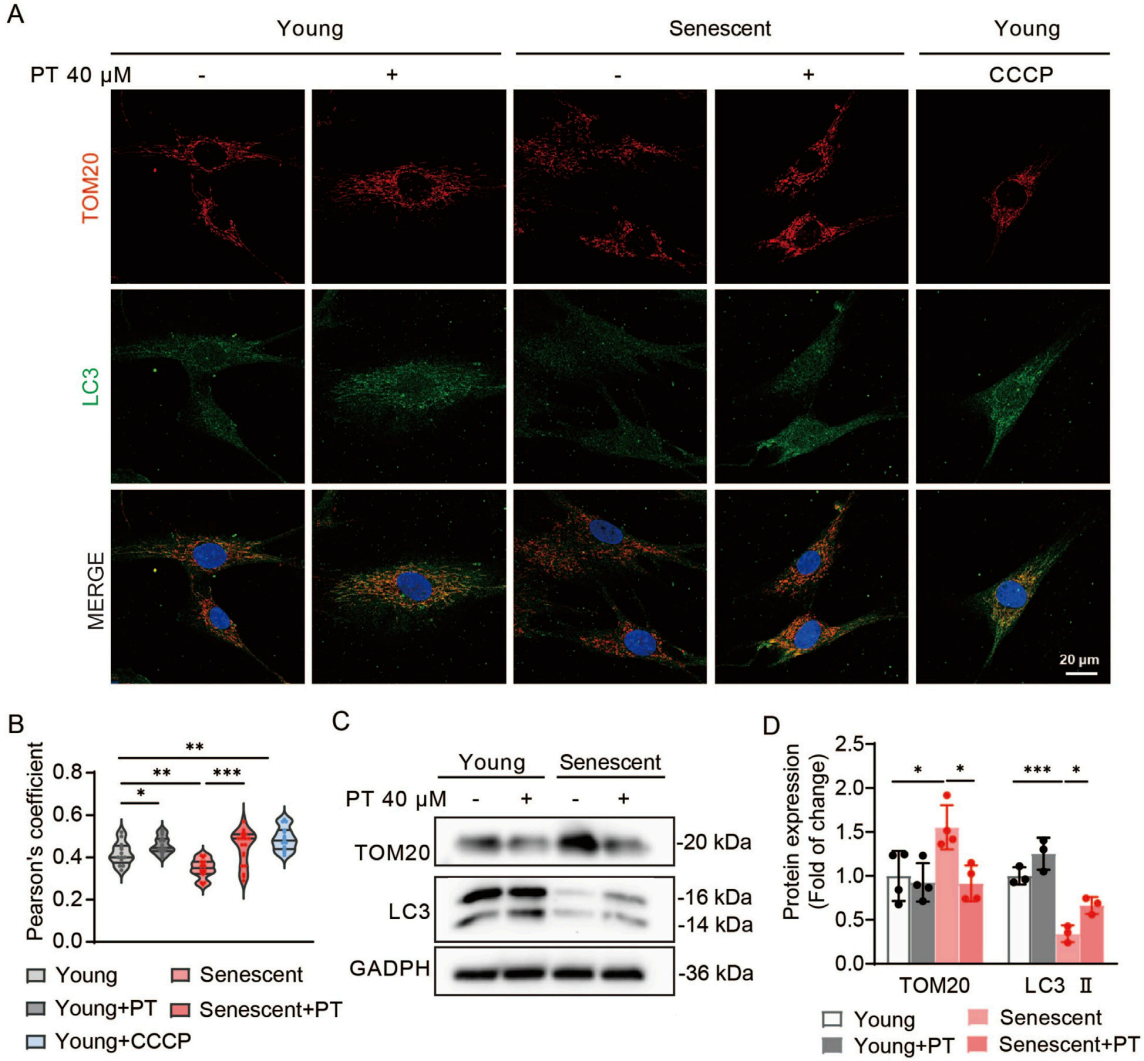
Effects of PT on mitophagy in replicative senescent HDFs. **(A)** Representative immunofluorescence images of co-staining with TOM20 and LC3 in HDFs. Cells in the CCCP group were treated with 10 μM CCCP for 30 min as a positive control. Scale bar = 20 μm. **(B)** Quantification of co-localization efficiency of Tom20 and LC3 (*n* = 13–19). **(C)** Western blot images and **(D)** quantification of TOM20 and LC3 protein expression in senescent HDFs (*n* = 4). Data were shown as mean ± SD of three independent experiments. Statistical significance was determined by two-way ANOVA with Tukey’s multiple comparisons test. ***p < 0.001, **p < 0.01, *p < 0.05.

Our previous results proved that PT can improve mitochondrial function and enhance mitochondrial quality control, in which mitophagy plays a key regulatory role. We evaluated PT’s impact on mitophagy in replicative senescent HDFs. Co-localization fluorescence images displayed a significant reduction in the co-localization efficiency in senescent HDFs, whereas PT treatment enhanced the co-localization efficiency both in young and senescent HDFs (Figures 5A,B). Western blot results revealed that the expression levels of LC3 Ⅱ were significantly reduced and the expression levels of mitochondrial proteins TOM20 were significantly increased in senescent HDFs, which was reversed by PT treatment (Figures 5C,D). Thus, mitophagy was impaired and damaged mitochondrial accumulated in senescent HDFs, while PT promoted mitophagy to remove damaged mitochondrial and maintained mitochondrial mass homeostasis.

### 3.6 PT prevented chronic UVB irradiation-induced skin damages

**FIGURE 6:**
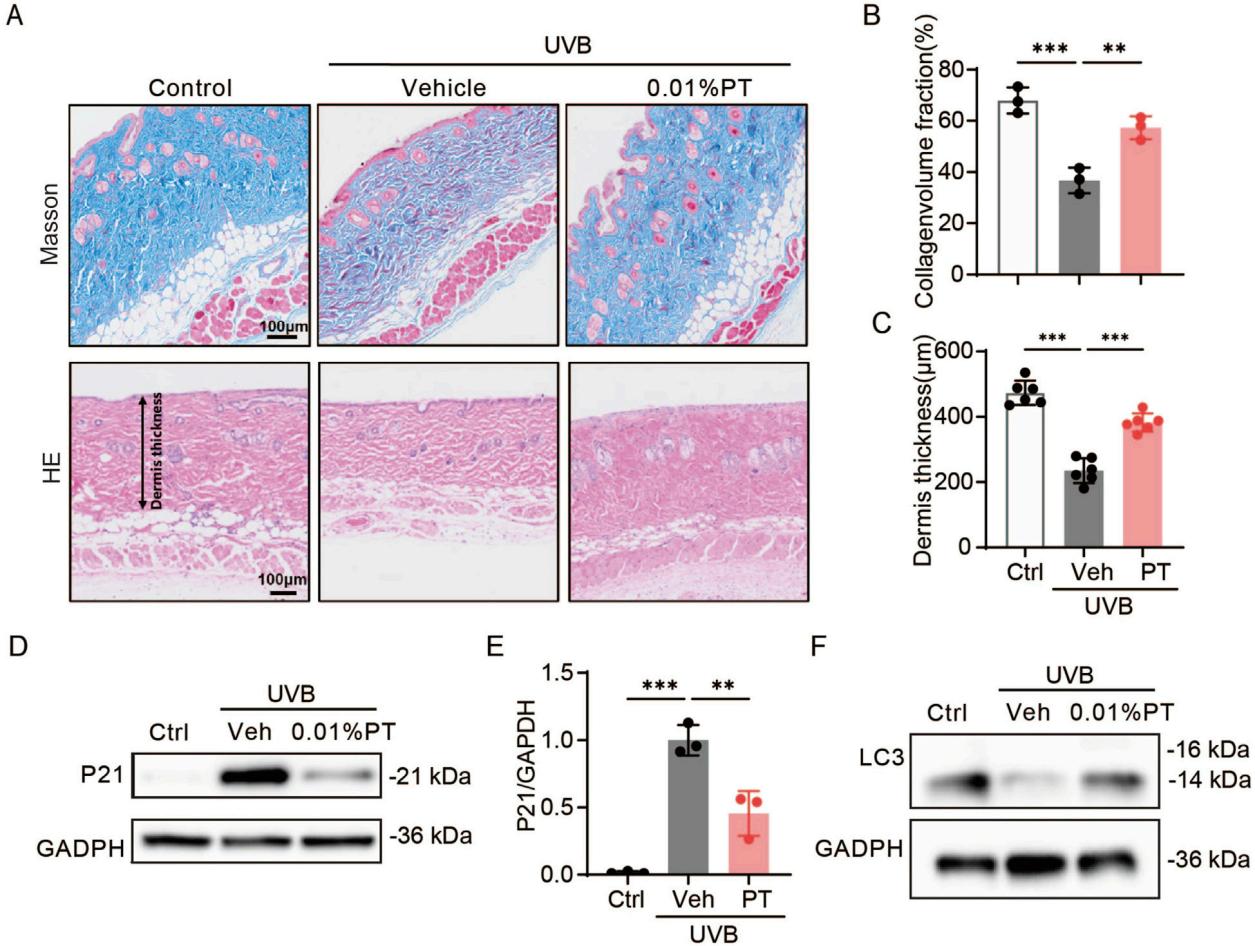
Effects of PT on the dorsal skin of UVB-irradiated mice. **(A–C)** Collagen density was assessed using Masson staining, and dermis thickness was evaluated using HE trichrome staining (*n* = 3–6). Scale bar = 100 μm. **(D)** Western blot images and **(E)** quantification of p21 protein expression in UVB-irradiated mice (*n* = 3). **(F)** Western blot images of LC3 protein expression in UVB-irradiated mice. Data were shown as mean ± SD of three independent experiments. Statistical significance was determined by one-way ANOVA with Tukey’s multiple comparisons test. ***p < 0.001, **p < 0.01.

HE and Masson staining revealed that UVB irradiation significantly decreased dermal thickness and collagen density in mouse skin compared to the control group. Importantly, long-term continuous PT application significantly reversed these UVB-induced damages (Figures 6A–C). Western blot analysis showed that PT treatment mitigated the UVB-induced upregulation of p21 protein level in mouse skin (Figures 6D,E), suggesting its protective effect against photoaging. Furthermore, PT application restored the protein levels of autophagy marker LC3, which were reduced by UVB exposure (Figure 6F). This restoration of LC3 levels suggests that the PT promotes autophagy, which is a potential mechanism through which PT exerts its anti-photoaging effects.

## 4 Discussion

Skin aging results from both intrinsic factors, such as replicative senescence, and extrinsic factors, including chronic UV exposure. Replicative senescence is primarily driven by telomere shortening and cumulative oxidative stress, leading to irreversible cell-cycle arrest and the secretion of senescence-associated secretory phenotype (SASP) factors that impair dermal structure and function (Woo et al., 2022; Zonari et al., 2023). In contrast, UVB irradiation accelerates photoaging by inducing DNA damage, ROS accumulation, and mitochondrial dysfunction (D'Orazio et al., 2013; Panich et al., 2016; Tak et al., 2024). In this study, we evaluated the anti-skin-aging effect of PT and investigated its potential molecular mechanisms. Two models of HDF senescence were established *in vitro*: a UVB-induced acute oxidative stress model and a replicative senescence model achieved through serial passaging. In both models, PT treatment effectively reversed aging-associated phenotypes in senescent HDFs. PT significantly reduced aging markers, including SA-β-gal, p16, and p21, while upregulating collagen Ⅰ and Ⅲ expression. Moreover, PT restored mitochondrial morphology, enhanced mitochondrial membrane potential, decreased ROS accumulation, increased ATP production and respiratory efficiency, and promoted mitophagy. *In vivo*, topical application of PT to UVB-exposed mouse dorsal skin restored collagen deposition and dermal thickness while reducing p21 protein expression. These findings demonstrate that PT delays HDF senescence and restores fibroblast functionality by improving mitochondrial quality, highlighting its promise as a novel therapeutic agent for skin aging.

PT, a methoxylated analog of resveratrol, exhibits pleiotropic pharmacological activities including anti-cancer, neuroprotective, anti-obesity, anti-diabetic and anxiolytic activities (Aguirre et al., 2016; Al et al., 2013; Nutakul et al., 2011; Poulose et al., 2015; Tastekin et al., 2018). In addition, studies have also demonstrated the photoprotective effect of PT on mouse skin (Muralitharan et al., 2025; Sirerol et al., 2015). PT antagonizes UVB-induced skin aging by simultaneously attenuating oxidative stress, inflammatory infiltration and immunosuppression (Sirerol et al., 2015). Its elevated lipophilicity enhances membrane permeation and potentiates Nrf2/ARE activation, thereby restoring antioxidant enzymes and glutathione while suppressing SASP mediators that degrade dermal ECM (Bhakkiyalakshmi et al., 2014; Chiou et al., 2011; Hseu et al., 2021). However, current studies on PT’s anti-skin aging effects remain limited with the UV-induced acute oxidative stress model being the sole employed system, and antioxidant activity representing the only consistently reported mechanism. We demonstrate for the first time that PT pre-treatment effectively rejuvenated senescent HDFs by targeting mitochondrial dysfunction and enhancing mitophagy. Our findings establish PT as a novel anti-aging intervention that targets both chronological and UV-driven skin aging.

Compared with retinoids, PT displays equivalent procollagen I restoration yet superior suppression of mitochondrial ROS without provoking erythema, desquamation, or photosensitivity (Griffiths and Voorhees, 1993). This superior performance and enhanced tolerance stem from PT’s methoxy groups, which significantly increase its lipophilicity and photostability. This structure modification leads to a 4- to 6-fold higher dermal delivery, thereby enhancing its bioavailability and therapeutic efficacy. Furthermore, PT’s Generally Recognized As Safe (GRAS) status and non-teratogenic properties allow for pregnancy-safe, long-term anti-aging use, addressing key limitations of retinoids (Yeo et al., 2013). Collectively, these attributes position PT as a compelling, pregnancy-safe, non-irritating, and highly efficacious alternative to topical retinoids for long-term anti-aging skincare.

Malfunctioning mitochondria can lead to a wide range of cellular problems, including increased oxidative stress and impaired energy metabolism (Zhou et al., 2024). Consistent with previous reports, increased mitochondrial ROS and decreased MMP indicated significant mitochondrial dysfunction in senescent fibroblasts (Passos et al., 2007). This study demonstrated that PT reversed structural and functional mitochondrial damage. In addition, the seahorse results indicated higher baseline mitochondrial activity in PT pre-treatment HDFs compared to senescent HDFs.

Mitochondrial dynamics, biogenesis, and mitophagy are crucial processes that coordinate to maintain mitochondrial homeostasis and restore damaged mitochondrial function (Nutakul et al., 2011). Accumulating evidence establishes mitochondrial dysfunction as a central driver of skin aging. Senescent dermal fibroblasts harbour hyper-elongated, functionally static mitochondria that generate excessive ROS, accumulate mtDNA and precipitate bioenergetic failure (Guo et al., 2023; Miwa et al., 2022; Tak et al., 2024). Impairment of the mitochondrial fission/fusion machinery coupled with declining mitophagy compromises mitochondrial quality control, accelerating skin aging (Archer, 2013; Smith and Carroll, 2025). Among these, mitophagy, the selective autophagic removal of damaged or excessive mitochondria, plays a pivotal role in mitochondrial quality control and energy homeostasis (Poulose et al., 2015). In this study, we observed impaired mitophagy and accumulated mitochondrial mass in senescence, and the accumulation of mitochondrial mass may be attributed to insufficient mitophagy. We demonstrated that PT significantly promoted mitophagy and improved mitochondrial quality in senescent HDFs. In addition, studies have reported that activated mitophagy acted as an emergency mechanism to prevent the accumulation of damaged mitochondria in the early stages of UV-induced senescence of HDFs (Cavinato et al., 2024; Chen et al., 2022). While PT was effective in both senescence models, subtle differences in the kinetics of reversal or the specific pathways predominantly modulated by PT were observed between replicative and UVB-induced senescence. The observed differences in responses between replicative and UVB-induced senescence emphasize the complexity of cellular aging and the need for tailored therapeutic strategies. In summary, our work provides novel theoretical evidence for PT’s anti-aging effects, highlighting the central role of mitochondrial quality control in cellular senescence, thereby establishing an experimental foundation for developing mitochondria-targeted anti-aging strategies.

## Data Availability

The original contributions presented in the study are included in the article/supplementary material, further inquiries can be directed to the corresponding authors.
